# Providers of relief in distress: RAG-based LLMs as situation and intent-aware assistants

**DOI:** 10.3389/frai.2026.1712596

**Published:** 2026-03-02

**Authors:** Ahmad M. Nazar, Brianna Norman, Halle Northway, Abrahim Toutoungi, Emma Zatkalik, Gabriel Carlson, Ellery Sabado, Hamza Shawa, Mohamed Y. Selim

**Affiliations:** 1 Department of Electrical and Computer Engineering, Iowa State University, Ames, IA, United States; 2 Gladiolus Technological Institute, Ames, IA, United States; 3 Department of Psychology, Iowa State University, Ames, IA, United States

**Keywords:** AI agents, generative AI, health assistant, LLMs, machine learning, retrieval-augmented generation

## Abstract

In high-stress humanitarian and mental health contexts, timely access to accurate, empathetic, and actionable information remains critically limited, especially for at-risk and underserved populations. This work introduces LLooMi, an open-source, retrieval-augmented generation (RAG) conversational agent designed to deliver trustworthy, emotionally attuned, and context-aware support across domains such as mental health crises, housing insecurity, medical emergencies, immigration, and food access. Leveraging large language models (LLMs) with structured prompting, LLooMi reformulates user queries into actionable intents, often implicit, emotionally charged, or vague. It then retrieves and grounds responses in a curated, domain-specific knowledge base, without storing personal user data, aligning with privacy-preserving and ethical AI design principles. LLooMi adopts an intent-aware architecture that adapts its tone, content, and level of detail based on the user's inferred psychological state and informational goals. This step enables delivering fast, directive responses in acute distress scenarios or longer, validation-oriented support when emotional reassurance is needed, emulating key facets of therapeutic communication. By integrating NLP-driven semantic retrieval, structured dialogue memory, and emotionally adaptive generation, LLooMi offers a novel approach to scalable, human-centered digital mental health interventions. Evaluation shows an average answer correctness (AC) of 92.4% and answer relevancy (AR) of 84.9%, with high scores in readability, perceived trust, and ease of use. These results suggest LLooMi's potential as a complementary NLP-based tool for mental health support in digital psychiatry and crisis care.

## 1 Introduction

Integrating Artificial Intelligence (AI) into digital health ecosystems reshapes how individuals access critical support across medical, legal, and social domains. Large language models (LLMs) are deep neural networks trained on vast and heterogeneous corpora at the forefront of this transformation. LLMs enable open-domain, conversational interaction grounded in context and semantics (Chang and Bergen, 2024; Celik and Eltawil, 2024). Unlike traditional rule-based or task-specific models, LLMs offer scalable, real-time, and generalizable assistance, making them especially promising in complex, under-resourced scenarios such as mental health crises, immigration processes, housing instability, and food insecurity (Brown et al., 2020; Radford et al., 2019; Zheng et al., 2023; Thirunavukarasu et al., 2023).

Previous generations of digital health systems primarily leveraged supervised machine learning (ML) or rule-based expert systems to power discrete functionalities such as diagnostic triage, symptom checking, or electronic health record management (Rajkomar et al., 2019; Plaat et al., 2025). While these systems demonstrated utility within tightly defined tasks, they often lacked contextual reasoning, dialogue coherence, and the ability to dynamically adapt to a user's evolving emotional or informational state. Advances in natural language processing (NLP) and deep learning have broadened the conversational capabilities of such systems (Chen et al., 2017). However, many remain domain-constrained (e.g., single-purpose mental health chatbots) and do not generalize across the interconnected challenges faced by marginalized or vulnerable populations.

LLMs offer a general-purpose language understanding framework, allowing for fluid transitions between domains and deeper user intent modeling. However, their deployment in health and humanitarian settings raises significant risks. These models are inherently probabilistic and non-deterministic, leading to hallucinations, overconfident misstatements, or misinterpreting ambiguous queries (Xu et al., 2024; Gao et al., 2023). Consequently, responsible use of LLMs in high-stakes environments demands the incorporation of ethical guardrails, verifiable grounding, and explicit disclaimers to ensure safe and trustworthy operation.

To address these challenges, we introduce LLooMi, a retrieval-augmented, empathetic digital assistant designed to support a broad spectrum of health and humanitarian queries. LLooMi combines a retrieval-augmented generation (RAG) framework with structured knowledge bases and memory-aware orchestration to provide factually grounded, context-sensitive responses across eight core domains, including medical, legal, food, housing, immigration, and crisis intervention (Lewis et al., 2020; Gao et al., 2023; Huang et al., 2025). Rather than relying on black-box LLM outputs, LLooMi conditions generation on verifiable external knowledge sources, reducing hallucination risk while improving response specificity.

A defining feature of LLooMi is its empathy-aware response model. Instead of relying on fine-tuned sentiment-labeled datasets, LLooMi uses primed instruction templates to guide LLM behavior in emotionally sensitive contexts (Chen et al., 2024). These templates simulate compassionate tone and provide behavioral constraints appropriate to domains such as trauma care, mental health, and abuse support, enabling emotionally intelligent responses without modifying the base model (Großmann et al., 2025). Moreover, LLooMi explicitly avoids diagnostic claims or prescriptive legal guidance and includes dynamic safety messaging, e.g., directing users to emergency services such as 911 when necessary.

Several contemporary systems highlight the growing role of LLMs in digital health. For instance, the World Health Organization's HealthBuddy+ chatbot provided localized public health guidance during the COVID-19 pandemic (World Health Organization, 2022). Domain-specific models like MedGPT (Kraljevic et al., 2021) and FoodBot (Prasetyo et al., 2020) have showcased the benefits of fine-tuning LLMs for medical and nutrition support, respectively. However, these systems remain task-specific and siloed, often lacking the cross-domain generalization and emotional contextualization needed in humanitarian scenarios.

LLooMi aims to bridge the gap between factual accuracy and emotional relevance in high-stakes humanitarian and digital health scenarios by delivering a unified, adaptable, and ethically grounded assistant. At its core, LLooMi leverages an intent-aware architecture that dynamically adapts responses based on the inferred urgency, tone, and informational needs of the user, allowing it to respond with conciseness in crisis contexts or with reassurance and validation when emotional support is needed. This flexibility is achieved through carefully engineered system prompts and a structured RAG pipeline, without relying on fine-tuned models or invasive user tracking.

To support a wide range of user needs in humanitarian and healthcare contexts, LLooMi is built on a comprehensive multi-domain assistance base, composed of eight distinct datasets spanning critical domains: housing, food security, immigration, financial assistance, LGBTQ+ support, sexual health, general medical care, and mental health. Each dataset was manually curated from authoritative sources to ensure high-quality, regionally relevant, and actionable content. To enhance factual grounding and emotional alignment, each domain is paired with a custom instruction prompt template that tailors the assistant's behavior, governing what and how it is said. These templates help LLooMi adapt tone, content structure, and urgency based on the domain and inferred user intent, ensuring responses are informative, emotionally appropriate, and context-sensitive.

Moreover, LLooMi's architecture prioritizes user experience and accessibility, incorporating elements such as prompt formatting constraints for readability, anonymous deployment to protect user privacy, and a lightweight system that can run without requiring cloud-based fine-tuning or sensitive data collection. Evaluations demonstrate that LLooMi achieves up to 92.4% average response correctness and 84.9% average relevancy. These results illustrate LLooMi's potential as a scalable, trustworthy, and user-centered AI solution for navigating real-world crises with competence and care.

## 2 Materials and methods

### 2.1 Overview of LLooMi

**Figure 1:**
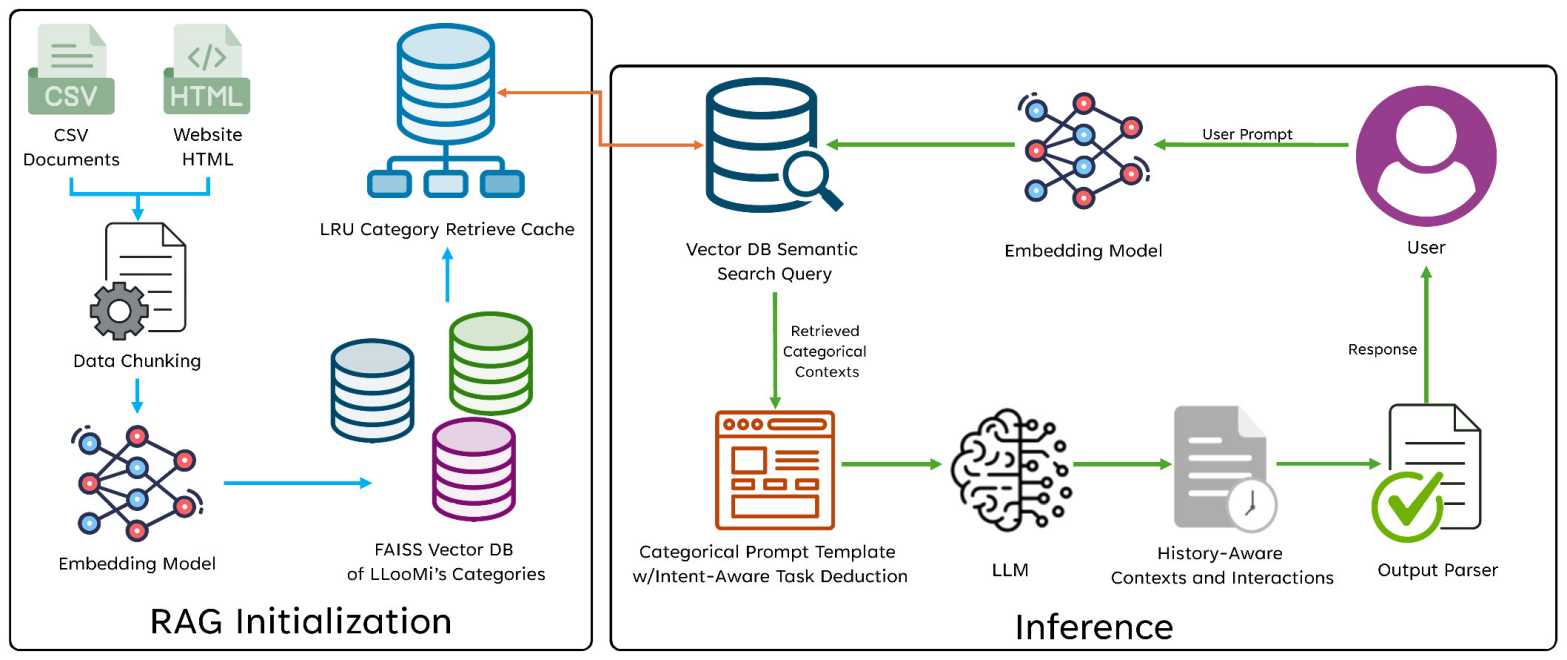
LLooMi's general flow and system architecture.

LLooMi is a humanitarian-focused assistant that provides real-time, context-sensitive guidance across various health and support domains. An overview of the internal architecture of LLooMi is seen in Figure 1. Powered by an LLM, the LLooMi system operates using a RAG pipeline. It begins with ingesting a curated, multi-domain dataset from verified public, governmental, and nonprofit sources such as the Red Cross, Feeding America, the Department of Housing and Urban Development, and more. The dataset includes critical topics such as medical emergencies, mental health, housing, food security, immigration, financial aid, LGBTQ+ resources, and sexual health. All information is organized into structured CSV files, where each file represents a specific topic and U.S. state pairing, and includes fields such as contact information, service descriptions, and areas of operation.

The dataset is supplemented with dynamic web scraping to ensure that information is current to include updates on crisis resources, program changes, and regional availability. The collected data is then segmented into smaller, overlapping text chunks. These chunks are converted into dense, numerical vector embeddings using a general text embeddings (GTE) model. This model maps semantically similar pieces of information close together in a high-dimensional space. These embeddings are stored in a vector database that can be indexed for efficient similarity search.

When a user submits a query, LLooMi employs a context-aware retrieval method to generate a response. It leverages the full conversation history to reformulate and further supplement the query for a more well-rounded LLM response. This step ensures that vague, emotional, or multi-turn queries are transformed into more precise and structured requests. This rewritten query performs a semantic search against the vector database, retrieving and ranking to select most relevant chunks of data based on the users' intent, context, and domain specificity.

Finally, the retrieved information, alongside the original query and chat history, is passed into a domain-specific RAG prompt with a task deduction and criteria for intent-aware response generation. Each domain has an instruction template, such as an empathetic tone for mental health or a directive clarity for medical first aid. This template ensures that the prompt's response is accurate, grounded, and emotionally attuned to the user's needs. Once a response is generated, the situation-aware response is processed through an output parser to be displayed in a user interface and delivered back to the user.

LLooMi avoids hallucinations by relying on a static, human-verified knowledge base with version control and safety disclaimers. It uses instruction priming rather than emotional fine-tuning to stimulate compassion. This priming allows LLooMi to operate efficiently even on resource-constrained machines, delivering personalized, safe, and reliable guidance across various humanitarian scenarios.

In the following sections, we break down the system architecture that LLooMi operates on into more detail.

### 2.2 Dataset

We curated a comprehensive multi-domain dataset from trusted public, governmental, and nonprofit sources to ensure LLooMi delivers accurate, relevant, and context-sensitive assistance across a broad range of humanitarian and health-related scenarios. The dataset covers critical support areas, including medical emergencies, mental health, food insecurity, housing, immigration, financial aid, LGBTQ+ resources, and sexual health. All sources were carefully reviewed and manually structured to enable effective retrieval through the system's RAG pipeline.

Information was extracted and organized into structured CSV files, enabling rapid indexing and semantic retrieval. Data fields include organization names, service descriptions, eligibility criteria, operating hours, contact information, addresses, and regional relevance. This structured format ensures that LLooMi can respond with specific and actionable information tailored to user needs and local context.

An overview of the dataset's primary domains, sources, and representative services is summarized in Table 1. Below, we provide brief descriptions that highlight the depth and diversity of each domain:

- **Medical:** Includes emergency symptoms (e.g., chest pain, seizures), first-aid protocols, and chronic disease guidelines. Data were sourced from the American Red Cross, Mayo Clinic, and various state Department of Health and Human Services (DHHS) portals.
- **Mental health:** Covers crises such as suicidal ideation, anxiety, and substance use. Sources include National Alliance on Mental Illness (NAMI), the Substance Abuse and Mental Health Services Administration (SAMHSA), and the American Foundation for Suicide Prevention (AFSP), with emphasis on helplines, peer support, and referral pathways. Resources include helplines (e.g., 988 Suicide & Crisis Lifeline), peer support networks, and referrals to state and local mental health services.
- **Food assistance:** Focuses on food banks, SNAP/WIC eligibility, and nonprofit food access. Data were drawn from Feeding America, local United Way chapters, and state Human Services Departments. For instance, Feeding America's nationwide food bank directory is indexed to allow region-specific referrals.
- **Housing:** Provides information on emergency shelters, rent relief, and housing legal aid, sourced from U.S. Department of Housing and Urban Development (HUD), local housing authorities, and shelter directories like Homeless Shelter Directory. LLooMi can assist users in identifying nearby shelters, understanding eligibility for housing aid, or connecting with legal housing support services.
- **Immigration:** This category supports users navigating visa applications, asylum processes, work permits, and documentation needs. Information is sourced from the U.S. Citizenship and Immigration Services (USCIS), Immigration Advocates Network, and nonprofit legal clinics such as Refugee and Immigrant Center for Education and Legal Services (RAICES). These entries help users access multilingual legal aid, immigrant rights resources, and documentation checklists.
- **Financial:** Covering federal and state-level support programs, this dataset includes content from Federal Student Aid (FAFSA), the Department of Education, Temporary Assistance for Needy Families (TANF), and state revenue departments. It also includes nonprofit financial aid directories for rent relief, emergency cash assistance, and utility support.
- **LGBTQ+:** This domain provides access to helplines, safe spaces, legal advocacy, mental health support, and community-building resources for LGBTQ+ individuals. Sources include The Trevor Project, the LGBT National Help Center, and NAMI's LGBTQ+ youth programming. For example, LLooMi uses this dataset to guide users to identity-affirming mental health resources, legal help for discrimination cases, or community organizations.
- **Sexual health:** Focused on sexual health education, sexually transmitted infection (STI) prevention, and survivor support, this dataset includes content from Planned Parenthood, state DHHS sexual violence coalitions, and the National Sexual Assault Hotline (RAINN). It enables LLooMi to connect users with safe houses, crisis centers, and confidential medical resources.

**Table 1:** Summary of dataset domains, sources, and example services used in LLooMi.

**Category**	**Main sources**	**Example services**
Medical	American Red Cross, Mayo Clinic, U.S. State DHHS portals	Emergency symptom triage, first-aid protocols, chronic disease self-care.
Mental health	NAMI, SAMHSA, AFSP	Crisis helplines (e.g., 988), peer support, local mental health referrals.
Food assistance	Feeding America, United Way, State Human Services Depts.	Food bank locators, SNAP/WIC eligibility, nonprofit food distribution.
Housing	HUD, Local Housing Authorities, Homeless Shelter Directory	Emergency shelters, rent assistance, housing advocacy.
Immigration	USCIS, RAICES, Immigration Advocates Network	Visa/asylum resources, multilingual legal aid, documentation checklists
Financial aid	FAFSA, TANF, Dept. of Education, State Revenue Depts.	Emergency cash aid, utility relief, rent and education assistance
LGBTQ+ Resources	The Trevor Project, LGBT National Help Center, NAMI LGBTQ+ Programs	Identity-affirming care, anti-discrimination legal aid, and support networks.
Sexual health	Planned Parenthood, State DHHS coalitions, RAINN	Sexually transmitted infection prevention, survivor support, confidential clinics and hotlines.

All datasets were manually curated to prioritize relevance, credibility, inclusivity, and regional coverage. Where possible, LLooMi surfaces resources based on the user' location or stated needs, while also including safety disclaimers for sensitive topics. We highlight that LLooMi is not a referral engine for trusted services and does not offer actionable clinical or legal advice, but acts as an initial source of assistance.

### 2.3 Data preprocessing

To ensure high-quality and localized information, we preprocess data through a dual approach that consists of structured CSV files and dynamic web scraping. Data preprocessing provides the benefit of enhancing a model's reliability (Maharana et al., 2022). Each dataset is first organized into individual humanitarian support categories, such as housing, mental health, sexual health, etc. The data is further segmented by U.S. state.

Each CSV file represents a specific topic-state pair and follows a consistent schema, with header fields such as: Website/Program Name, Program Type, URL, Address, Contact Information, Service Areas, Offered Services, and Purpose/About. These static CSV files serve as a foundational, curated knowledge base for LLooMi to build upon.

We utilize LangChain's WebBaseLoader to extract and parse content from web URLs to complement this structured data. This extraction enables real-time access to updates such as new services, program expansions, or contact changes, which is critical for crisis-related events.

Our static sources allow us to maintain structured, clean data, which works best for information such as contact information and locations of resources. Additionally, our dynamic sources allow access to up-to-date information such as new resources or events. All static and dynamic documents are then passed into the rest of the system, where they will be chunked into more workable sections and embedded into a vector database for analysis.

### 2.4 Data chunking

After preprocessing input data, the next step in LLooMi's pipeline involves data chunking. Preprocessed and structured data is split into smaller and equal-sized segments called chunks. RAGs and LLMs have limitations on how many tokens they can receive and process, and as such, segmenting large data into coherent and logical chunks improves the efficiency in processing, storing, and retrieving relevant data on inference (Bhat et al., 2025).

To ensure coherence and relevance of each chunk, there is an overlap of *c*_*o*_ characters between chunks to provide context in case of fragmented chunks. Taking advantage of chunk overlap further augments this process. This process is especially significant for data like multi-step instructions, where context potentially spans across multiple chunks. We split our data into chunk sizes *C*_*s*_ = 500 characters with an overlap of *c*_*o*_ = 100 characters. Based on our empirical tests within the pipeline, the selected chunk size and overlap efficiently utilized the embeddings model on vectorization and embedding (as explained in the next sub-section), and the fluidity of the generated LLM.

### 2.5 Assistance base creation

To support LLooMi's RAG pipeline, a structured, multi-domain assistance base that functions as a knowledge repository was developed as the foundation for semantic search and response generation. This base comprises high-quality, manually curated documents extracted from authoritative sources across eight key humanitarian and health-related domains, as described in Section 2.2. These sources include national health departments, nonprofit organizations, legal aid directories, and emergency service networks.

To ensure the knowledge base remains up-to-date in practical deployments, an automated background job periodically re-scrapes the original data sources referenced in Section 2.2, reprocessing and refreshing the assistance base as new information becomes available.

After data collection and preprocessing into structured formats (e.g., CSV, JSON), documents are chunked into smaller units of text, typically ranging between 100 to 300 words, depending on semantic boundaries and formatting heuristics. Chunking enables the system to preserve local context and retrieve only the most relevant segments rather than complete documents, thereby improving precision and reducing noise during query-time retrieval (Bhat et al., 2025).

Each chunk is transformed into a numerical vector embedding using a GTE model. Embeddings are high-dimensional dense vectors that capture the semantic content of the text. In this latent space, semantically similar text chunks are mapped closer together, while unrelated or off-topic chunks are embedded farther apart. This mechanism enables the system to match user queries not by surface-level keywords but by semantic proximity, enhancing LLooMi's ability to understand user intent even when queries are vague, emotional, or non-expert phrasing.

The resulting embeddings are stored in a vector database, indexed for approximate nearest-neighbor search. During inference, a user query is embedded using the same model and compared against the stored embeddings to identify the top-*k* (*k* = 95%) most relevant chunks. These chunks are then appended to a prompt primed with a domain-specific instruction template (e.g., empathetic tone for mental health, directive language for medical first aid) and passed to the LLM to generate a grounded, context-sensitive response as explained in the next section.

To reduce memory overhead and improve inference latency, especially when working with large or regionally partitioned knowledge bases, LLooMi implements a Least Recently Used (LRU) cache mechanism (Mayer and Richards, 2025). Each domain and region-specific knowledge base is preprocessed and stored in serialized vector formats. Only the most recently accessed vector indexes are retained in active memory upon user query, while less frequently accessed domains are unloaded or swapped from disk. This retention ensures that system memory is used efficiently and avoids repeated re-initialization of the same vector datasets, which is particularly beneficial when deployed on resource-constrained virtual machines or edge devices.

The assistance base is intentionally static and human-verified instead of relying on live web searches or user-generated content. This design minimizes the risk of LLM hallucinations caused by noisy or unvetted sources and allows LLooMi to operate within a tightly controlled and auditable knowledge environment. All updates to the assistance base are version-controlled and manually reviewed for factuality, inclusion, and ethical alignment.

Although LLooMi provides emotionally attuned responses, it does not rely on fine-tuning the LLM with emotion-labeled or empathy-specific training data. Instead, using carefully crafted domain-specific system prompts, empathetic behavior is achieved through instruction priming. These prompts are designed to elicit compassionate, supportive, and non-judgmental responses from the model, particularly in sensitive domains such as mental health and sexual health. This lightweight approach allows LLooMi to simulate emotional awareness while maintaining full generalization capability and minimizing the risks of model overfitting or unintended behavior.

This pipeline, composed of human-curated documents, semantically indexed embeddings, modular vector storage, and template-based LLM priming, enables LLooMi to deliver reliable, relevant, and sensitive assistance across a diverse set of user needs while remaining efficient, interpretable, and ethically grounded.

### 2.6 Retrieval

When a user submits a query to LLooMi, the system begins by capturing and structuring the ongoing conversation history. This structure includes the sequence of previous user inputs and model responses, concatenated and formatted into a rolling dialogue context. Maintaining this history is critical for preserving continuity and intent, especially when the conversation unfolds over multiple turns or involves clarifications, follow-ups, or shifting emotional states.

If a user is new or initiates a session without prior history, LLooMi initializes a default context by parsing the initial prompt as thoroughly as possible to extract relevant user intent, urgency, and emotional framing. As the dialogue progresses, each user input is appended to the session context, enabling LLooMi to build a dynamic understanding of the user's situation incrementally. This adaptive mechanism ensures that, even in first-contact scenarios, the system can begin tailoring its responses without extensive back-and-forth. The context accumulation forms an accessible chain throughout the session and serves as a backbone for all downstream retrieval and generation.

Each domain is paired with a domain-specific prompt template that modulates the tone, structure, and content of the response. For instance, if the conversation falls within the “mental health” domain, the system automatically activates a prompt that prioritizes confidentiality, empathetic language, and assurance-based response strategies. This behavior ensures that the retrieved content and generated outputs are factually relevant and emotionally and contextually appropriate.

Rather than treating each query as a standalone input, LLooMi leverages the accumulating dialogue to infer deeper conversational goals and preserve context awareness. This approach improves disambiguation, enables more personalized responses, and avoids repetition of previously provided information. The structured history is passed to a history-aware retriever powered by an LLM, which is used not for final response generation but as a query reformulator. This mechanism supplements the retrieval process by rewriting the user's input in light of the whole conversation context, yielding more precise and targeted retrieval queries.

The retriever performs this rewriting to convert the user's often implicit, vague, or emotionally framed messages into structured, actionable search queries. For example, an input like “I don't know what to do anymore” might be reformulated as “List available mental health crisis resources and helplines in the user's region.” This transformation ensures that even emotionally charged or imprecise user queries are reframed into information-seeking intents that can be meaningfully matched against the curated knowledge base.

Once the query is reformulated, LLooMi generates its semantic embedding using a lightweight GTE model. The resulting high-dimensional vector representation performs a semantic similarity search across domain-specific vector databases, each containing preprocessed, chunked, and embedded knowledge extracted from reliable external resources (see Section 2.5). Cosine similarity is used to quantify the semantic closeness between the embedded query and the stored document embeddings, allowing the system to surface conceptually aligned results even in the absence of keyword overlap.

To enforce high retrieval quality, the results are ranked by similarity score and filtered to retain only the top-*k* matching chunks. This ranking ensures that the documents passed to the generation step are highly relevant and contextually aligned with the user's current needs. Filtering out lower-ranking chunks reduces noise and hallucination risk while keeping the generated response grounded in high-confidence evidence.

Unlike traditional keyword-based retrieval systems, this semantic and history-aware pipeline allows LLooMi to surface documents that are topically appropriate and responsive to the user's emotional and situational context. For example, a distressed user referencing “no place to go” may still receive recommendations for local shelters, legal eviction help, and housing hotlines—despite not using technical terms.

Importantly, the history-aware retriever also helps prevent redundancy and over-retrieval. By incorporating prior conversation turns, the retriever is aware of what the user has already been told, reducing the chance of returning duplicate results or repeating prior information. This conversational memory enables smoother, less frustrating interactions and allows LLooMi to dynamically adjust what is retrieved based on what the user has already received.

This capability is especially critical in LLooMi's application domains, where users may be distressed, overwhelmed, or unable to articulate their needs clearly. The system's ability to reinterpret vague, indirect, or emotionally charged inputs and return relevant, supportive information is central to its mission as a human-centered AI assistant.

Through this integration of conversation history, structured query rewriting, GTE-based semantic embedding, cosine similarity filtering, and percentile-ranked document selection, LLooMi ensures its retrieval is context-sensitive, emotionally aware, and robust to ambiguity. This tightly integrated retrieval pipeline enables the generation module to operate with a rich and relevant informational foundation, ultimately improving user outcomes in real-world, high-stakes scenarios.

### 2.7 Intent-aware response generation

Once the relevant documents have been retrieved and ranked, LLooMi uses them as external resources to generate a response. The user query, the retrieved knowledge, and the complete chat history are passed into a structured RAG prompt that the LLM then processes. This pipeline does not rely on direct user profiling or fine-tuned emotion classifiers. Instead, it leverages structured prompts, accumulated conversation context, and domain-specific cues to guide the generation process.

Crucially, this architecture is intent-aware; LLooMi is designed to infer what the user is asking and why they are asking it. For example, in a housing-related query, the model distinguishes between a logistical request (“Where can I apply for Section 8?”) and an emotionally charged plea (“I was evicted and have nowhere to go”). In the first case, LLooMi generates a structured list of application links; the second prioritizes emergency shelters, free helplines, and immediate-action steps. Responses' tone, length, and format adapt accordingly: urgent queries prompt short, directive responses, while reassurance-seeking queries trigger longer, more validating outputs that embed facts and empathy.

Domain-specific prompt templates are central to LLooMi's behavior modulation, functioning as pre-configured instruction sets that define how the LLM should reason, structure, and communicate its responses within each category of user need. These prompts are dynamically activated based on the user's query's classified domain. They are engineered to encode procedural norms (how to answer) and empathic intent (how to frame the answer emotionally).

For example, in the “Medical” domain, the prompt primes the model to prioritize clarity and clinical safety, emphasizing symptoms, avoidance of speculative statements, and the inclusion of standard disclaimers and escalation pathways (e.g., “Call 911 if symptoms worsen”). This step ensures users receive swift, direct responses during time-sensitive situations. In contrast, a query under the “Immigration” domain activates a prompt that emphasizes step-by-step procedural guidance, detailed references to trusted legal sources (e.g., USCIS, RAICES), and a supportive tone calibrated to reduce anxiety or confusion. If a user asks, “I don't know what to do after my visa expires,” the system may respond with a checklist of legal aid resources, document requirements, and hotline contacts, presented with compassionate framing.

This type of prompt engineering enables the same base model to produce domain-specific response styles without altering its weights or architecture. It effectively acts as a lightweight control layer that adjusts reasoning depth, language tone, and information density based on the domain and inferred user emotional state.

These templates function like a soft emotional and procedural reasoning layer, allowing the model to modulate tone, pacing, and informational density based on inferred user state and domain requirements. Importantly, LLooMi achieves this without explicitly classifying emotion or intent; instead, it relies on primed context through prompt engineering, conversation framing, and structured input formatting. This design choice allows LLooMi to remain lightweight and interpretable while still delivering domain-appropriate behavior and responding to providing reassurance, validation, and detailed guidance whenever it is needed.

Finally, to ensure the system's outputs are presented in an accessible and user-friendly manner, an output parser post-processes the LLM output alongside the user query and full conversation history. This parser formats the response into a visually structured output, which is then rendered through a simple and intuitive user interface. This integration closes the loop between backend reasoning and front-end usability, allowing LLooMi to serve as both an empathetic agent and a usable tool across various crisis and assistance scenarios.

## 3 Results

### 3.1 Evaluation methodology

To comprehensively assess LLooMi's performance, we conducted a two-part evaluation: (1) a performative evaluation focused on response quality metrics, and (2) a user experience evaluation focused on usability, readability, and relevance as perceived by end users.

#### 3.1.1 Evaluation infrastructure and model setup

LLooMi was deployed on a high-performance server with an NVIDIA A40-8Q GPU (8GB VRAM), 32GB of RAM, and 1TB of local storage running Debian Linux. For language generation, LLooMi utilizes the LLaMa3.2-3B model (Dubey et al., 2024), selected for its balance between lightweight architecture and strong reasoning capabilities, allowing for efficient deployment and fast response times in resource-constrained settings. Inference with LLaMa3.2-3B consumes approximately 3.4GB of VRAM, leaving sufficient overhead for simultaneous retrieval and embedding operations.

To support semantic search, LLooMi employs the stella_en_1.5B_v5 GTE model (Zhang et al., 2025), chosen for its compact size and high-quality multilingual embeddings. All domain-specific datasets were preprocessed into vectorized chunks, totaling 14GB of storage and have been embedded into a Facebook AI Similarity Search (FAISS) vector database for native compatibility with semantic search mechanisms (Douze et al., 2024). During runtime, a LRU caching mechanism maintains up to 4GB of the most recently accessed domain-specific embeddings in memory, enabling faster multi-domain retrieval and efficient memory usage. This configuration ensures that LLooMi remains responsive and stable across diverse user interactions, even under moderate hardware constraints.

#### 3.1.2 Evaluation metrics for LLooMi's responses

For the performative evaluation, we adopted two quantitative metrics for evaluating generative LLM outputs: Answer Correctness (AC) and Answer Relevancy (AR). We used the LangSmith evaluation framework to automate the scoring of LLooMi's responses across controlled test prompts. To ensure domain fidelity, we constructed example datasets for each of LLooMi's eight supported domains, comprising 120 input prompts. Each dataset included reference human-generated question-answer pairs formatted in JSON and derived directly from the structured CSV files used to build the domain-specific assistance base. A sampling of these evaluations and outputs can be seen in Table 2. This evaluation ensured alignment between the RAG-based retrieval system and the curated ground truth resources. AC and cosine similarity for AR are commonly used standards in evaluating LLM-generated responses, as they jointly capture factual accuracy and semantic alignment between the system output and reference answers.

**Table 2:** Examples of prompts, their ground truths, variations of good and bad responses from LLooMi, and their AC and AR scores distress testing from the sexual health, mental health, and medical categories.

**Question**	**Ground truth**	**LLooMi answer**	**AC (%)**	**AR (%)**
What is the website for finding sexual assault programs in Iowa?	The website for finding sexual assault programs in Iowa is https://www.iowacasa.org/get-help.	In Iowa, you can find information on sexual assault programs through the following websites: 1. National Sexual Assault Hotline (phone number) - For confidential support 2. Iowa Coalition Against Domestic Violence and Sexual Assault (ICADVA) - A Coalition to support sexual assault survivors.	90%	100%
How can someone contact the 988 Lifeline?	Someone can contact the 988 Lifeline by calling, texting, or chatting at 988.	You can contact the 988 Lifeline if you want. It is a phone number for mental health help. You can probably call it or look it up online. They help people who are having problems.	43%	47%
What services does the American Red Cross of Iowa provide?	The American Red Cross of Iowa provides services including disaster relief, first aid, and CPR across Iowa.	The American Red Cross of Iowa is a vital resource for individuals and families affected by disasters, emergencies, or everyday health concerns. With a presence in all 99 counties across the state, this organization provides essential services to those in need. To learn more about the services provided, you can visit their website: https://www.redcross.org/local/iowa/about-us.html	100%	100%

These test cases were hand-designed to reflect realistic usage scenarios and to assess whether LLooMi could retrieve the correct knowledge and generate an informative, contextually appropriate, and emotionally attuned response. These two components offer a holistic view of LLooMi's effectiveness in system-level accuracy and human-centered usability.

AC measures whether a generated response is semantically faithful and factually aligned with a predefined ground truth. It is computed as a weighted combination of semantic similarity and factual overlap. Semantic similarity is calculated using the cosine similarity between the embedding of the generated response (${\vec{E}}_{a_{i}}$) and the embedding of the reference answer (${\vec{E}}_{t_{i}}$). Factual overlap, on the other hand, is measured using an F1-style metric over extracted factual units:


$$\begin{array}{l} F=\frac{\left|\text{TP}\right|}{|\text{TP}|+0.5\left(\left|\text{FP}|+|\text{FN}\right|\right)}, \end{array}$$ (1)

where TP refers to factual statements in both the generated and reference responses, FP refers to hallucinated or extraneous facts, and FN denotes critical facts in the reference that are missing from the generated answer. The overall AC score is defined as:


$$\begin{array}{l} \text{AC}=\omega \cos\left({\vec{E}}_{a_{i}},{\vec{E}}_{t_{i}}\right)+\left(1-\omega \right)F, \end{array}$$ (2)

with ω = 0.25 in our experiments, reflecting our emphasis on factual fidelity over purely semantic alignment. The cosine similarity term measures semantic alignment between the generated response and the reference answer and theoretically ranges from −1 to 1. In practice, however, cosine similarity values for text embeddings used in LLM-based semantic search and retrieval tasks fall within the range [0, 1] and often clustering at higher values (e.g., 0.5–0.9). This behavior arises because natural language embeddings occupy similar directions in high-dimensional semantic space, making cosine similarity a reliable and widely adopted metric for evaluating semantic correctness in RAG systems.

This formulation allows us to distinguish between answers that are plausibly worded but factually incomplete and those that are both accurate and semantically aligned. AC was used in our evaluation to verify that LLooMi's answers included all essential details in reference answers from example datasets (e.g., LangSmith evaluations). Higher AC scores indicate strong agreement with ground truth responses, while lower scores indicate missing or hallucinated content.

AR complements this by measuring how well each generated response aligns with the user's original intent, independent of any reference answer. This metric is computed as the mean cosine similarity between the embedding of each generated response (${\vec{E}}_{a_{i}}$) and the embedding of the corresponding user query (${\vec{E}}_{t_{i}}$):


$$\begin{array}{l} \text{AR}=\frac{1}{N}\overset{N}{\underset{i=1}{\sum }}\cos\left({\vec{E}}_{a_{i}},{\vec{E}}_{t_{i}}\right)=\frac{1}{N}\overset{N}{\underset{i=1}{\sum }}\frac{{\vec{E}}_{a_{i}}\cdot {\vec{E}}_{t_{i}}}{\|{\vec{E}}_{a_{i}}{\|}_{2}\|{\vec{E}}_{t_{i}}{\|}_{2}}. \end{array}$$ (3)

In practice, this metric captures whether responses are factually correct and directly relevant to the user's question, even when the query is vague, emotional, or non-specific. A higher AR score indicates that the generated response effectively interprets and addresses the underlying query intent, while lower scores suggest off-topic or insufficiently responsive outputs. This metric is critical in LLooMi's deployment context, where users may struggle to precisely articulate their needs and require emotionally supportive or situation-aware assistance.

For AR, the grading criteria were based on how relevant LLooMi's answer was to the example question. This metric did not rely on the reference answers in the example dataset in LangSmith. For each answer generated from LLooMi, a grade between zero and one was given. A higher score indicated that the answer generated was highly relevant to the user's question, and a lower score indicated that the answer generated was not considered relevant to the question.

AC and relevance are equally important metrics to keep track of. Producing answers that have little to no relation to a query from a distressed individual is virtually useless and could put the user in even more distress. An answer that is not factually correct would be unhelpful to the user and potentially harmful if the given information in the answer is incorrect. Focusing on these two key criteria was the most important for our evaluation to display LLooMi's answer quality and trustworthiness.

#### 3.1.3 User study evaluation setup and metrics

For the user study, LLooMi was deployed to ten, general experience, volunteer participants. No personal information, identifiable data, or usage traces were collected from users, only their subjective feedback on the quality of the assistant. Participants were asked to rate their experience across four criteria: ease of use, readability of responses, relevance to their question, and overall satisfaction. Each dimension was rated on a scale from 1 to 10, and results were later normalized to percentage values to facilitate aggregate analysis. This study was approved by the Iowa State University Institutional Review Board (IRB), which determined that the research qualifies for exemption as it does not involve identifiable or sensitive information, and all participation was voluntary and anonymous.

### 3.2 Numerical results

**Figure 2:**
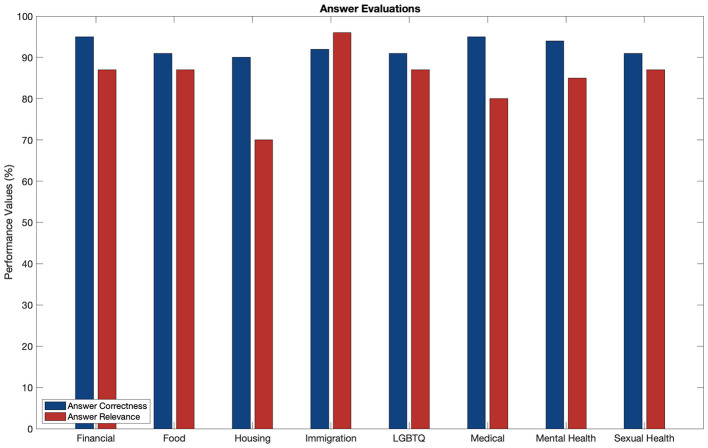
Performance of LLooMi's generated responses across AC and AR metrics within multiple dataset domains.

To evaluate LLooMi's effectiveness across both system performance and user satisfaction, we conducted a dual-layered assessment combining automated response metrics with direct user feedback. One hundred twenty domain-specific queries were used for the system-level evaluation, evenly distributed across LLooMi's eight supported categories. Each response was scored using the previously defined AC and AR metrics, capturing factual alignment and contextual relevance, as seen in Figure 2. On average, LLooMi achieved an AC score of 92.4%, with individual domains ranging from 90% in housing to 95% in medical and financial. These results indicate a strong ability to retrieve and convey accurate information grounded in the structured assistance base. For AR, LLooMi averaged 84.9%, with the highest scores observed in immigration (96%) and financial (87%). While most domains demonstrated high contextual alignment, housing exhibited a comparatively lower relevancy score of 70%, suggesting an opportunity for refinement in handling vague or under-specified user intents within that domain.

**Figure 3:**
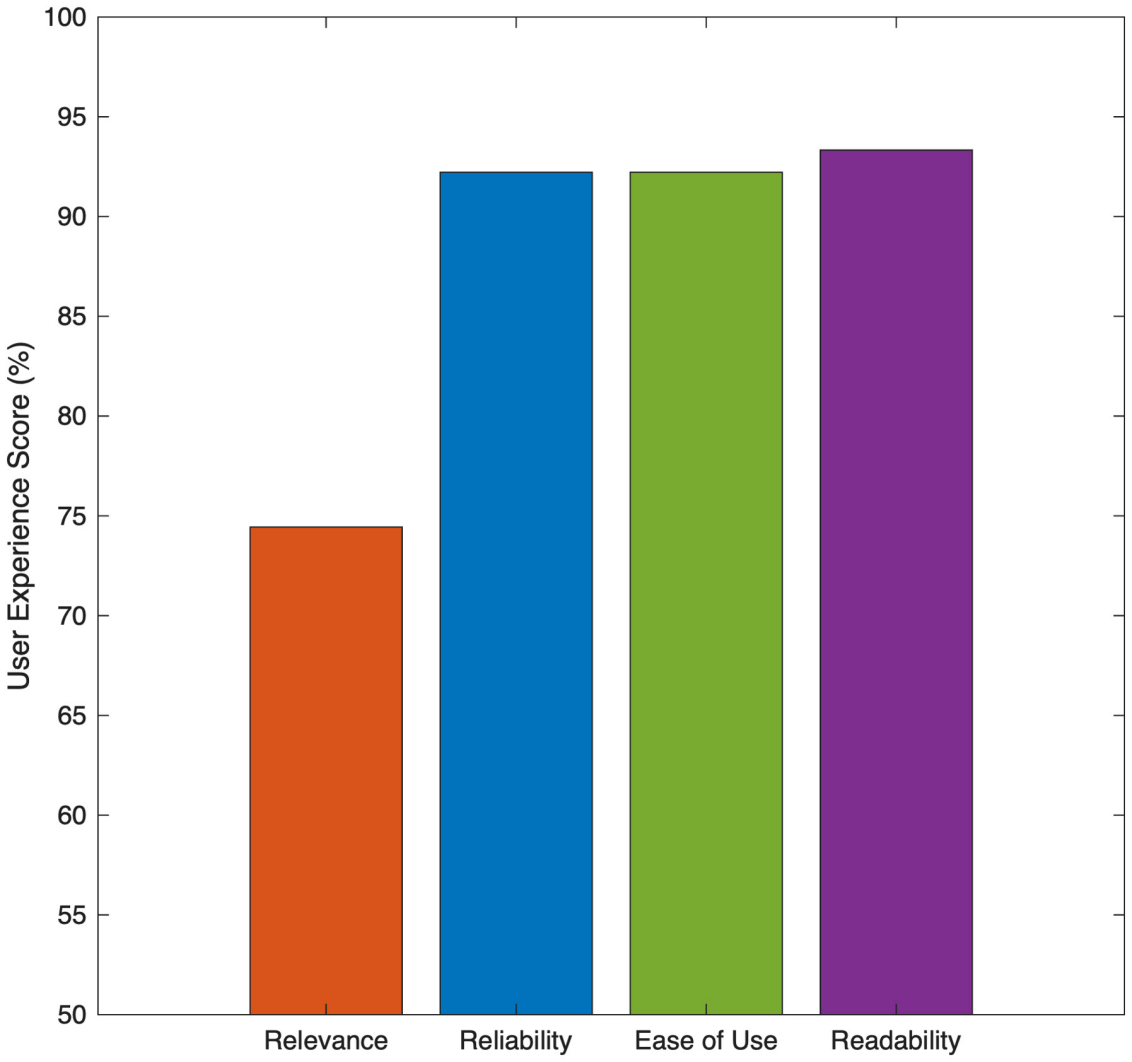
LLooMi's user experience results across different categories.

Complementing these automated evaluations, we conducted an anonymous, small-scale pilot study involving ten general experience participants who interacted with LLooMi in a controlled setting. While limited in scope, this initial user survey was designed to gather early insights into usability and perceived value, serving as a foundation for more comprehensive evaluations in future iterations. Participants were asked to rate four dimensions of their experience: relevance of responses, reliability of information, ease of use, and readability of outputs. Ratings were collected using a 10-point Likert scale and normalized to percentage values. As seen in Figure 3, the resulting scores were 92.22% for reliability and ease of use, 93.33% for readability, and 74.44% for perceived relevance. These results affirm that LLooMi was perceived as highly accessible, clear, and dependable. However, there is room for improvement in tailoring responses to individual perceptions of relevance, particularly when users expect personalized or hyper-localized recommendations.

These findings demonstrate that LLooMi performs robustly across both quantitative and experiential dimensions. The system exhibits firm factual grounding through its RAG architecture and maintains high user satisfaction through structured, emotionally attuned outputs. These results validate LLooMi's potential as an open-source, equitable health and humanitarian support digital assistant.

## 4 Case studies

### 4.1 Case study 1: optimal behavior

This case study highlights LLooMi's intended and optimal behavior when assisting users needing medical support. It demonstrates how LLooMi's retrieval-augmented architecture, empathy-primed response generation, and structured domain knowledge work in tandem to provide informative, emotionally aware, and actionable answers.

**Figure 4:**
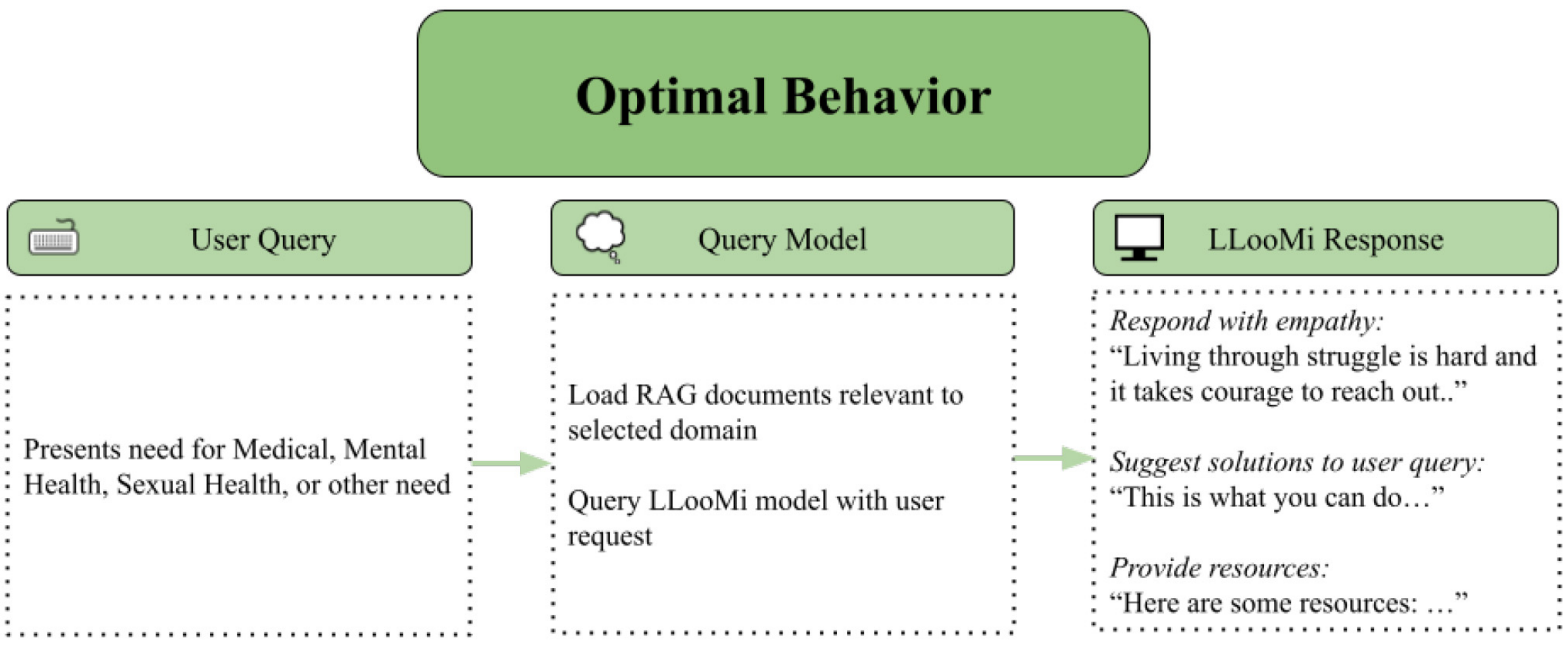
LLooMi's optimal behavior system flow from case study one.

**Figure 5:**
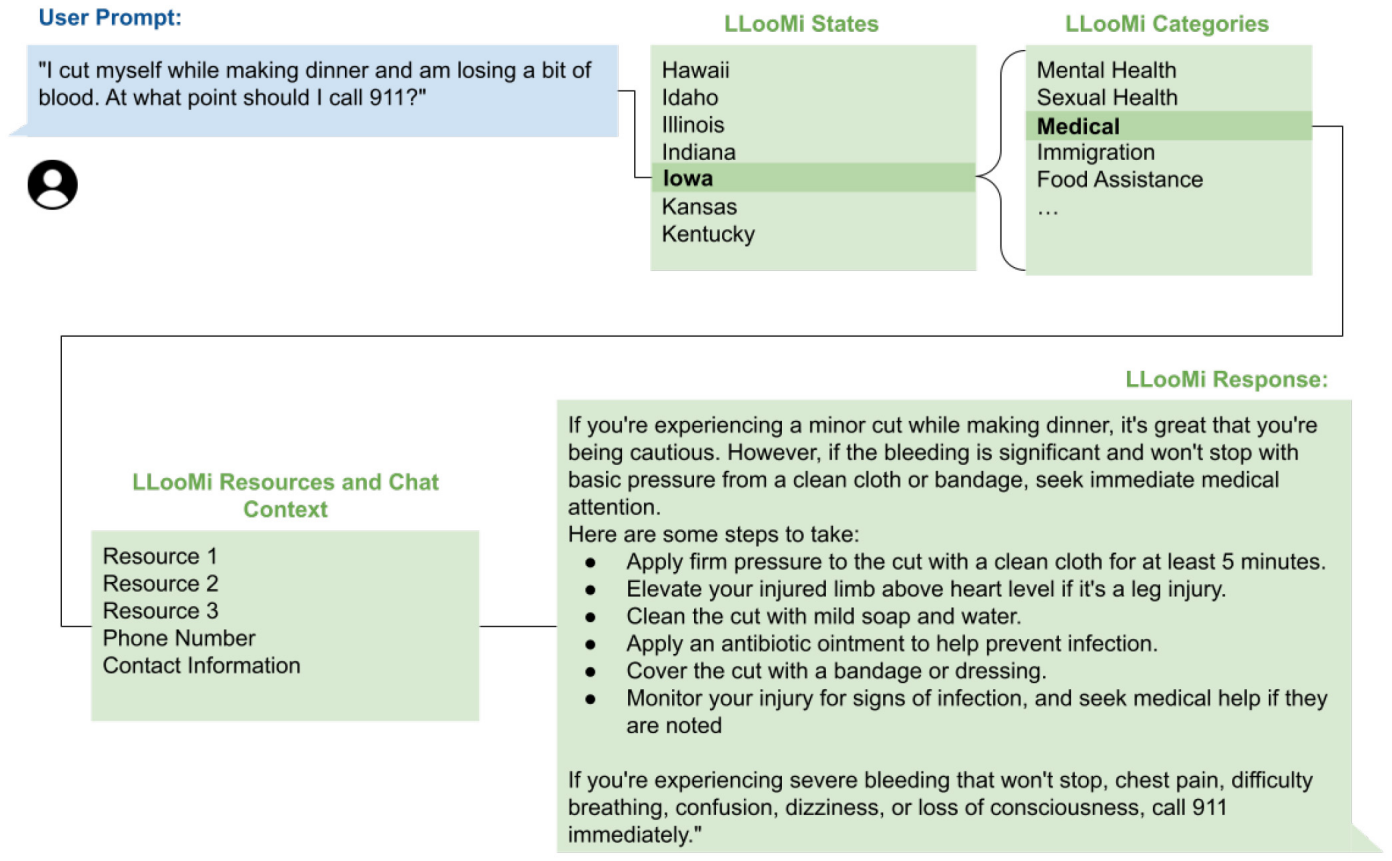
LLooMi's ideal response and how it retrieves information.

Figures 4, 5 illustrate a complete example in the medical domain. Figure 4 visualizes the internal LLooMi pipeline as it receives a user query, formats the prompt with prior conversation context, loads the appropriate domain-specific vector store (medical), and retrieves semantically relevant chunks from the curated assistance base. These retrieved entries are then passed to the LLM alongside a domain-specific instruction template that guides tone, structure, and safety messaging, where Figure 5 presents the resulting user interaction.

In this scenario, a user requests urgent medical support. LLooMi responds promptly with a direct answer, clearly structured next steps, and further advice. The system maintains a concise format while ensuring completeness, immediately addressing user needs, being clear in instruction, and following up with further things to monitor for. Importantly, LLooMi's response avoids generic language and centers the user's need with grounded, actionable steps.

LLooMi exemplifies its ability to synthesize domain knowledge and emotional context. It addresses ethical constraints (e.g., not offering diagnosis or legal advice) while demonstrating how an instruction-primed, retrieval-grounded LLM system can assist users with complex needs. These cases underscore LLooMi's core design principle: balancing factual accuracy, empathetic tone, and practical utility through modular, explainable AI pipelines.

### 4.2 Case study 2: comparative behavior

**Figure 6:**
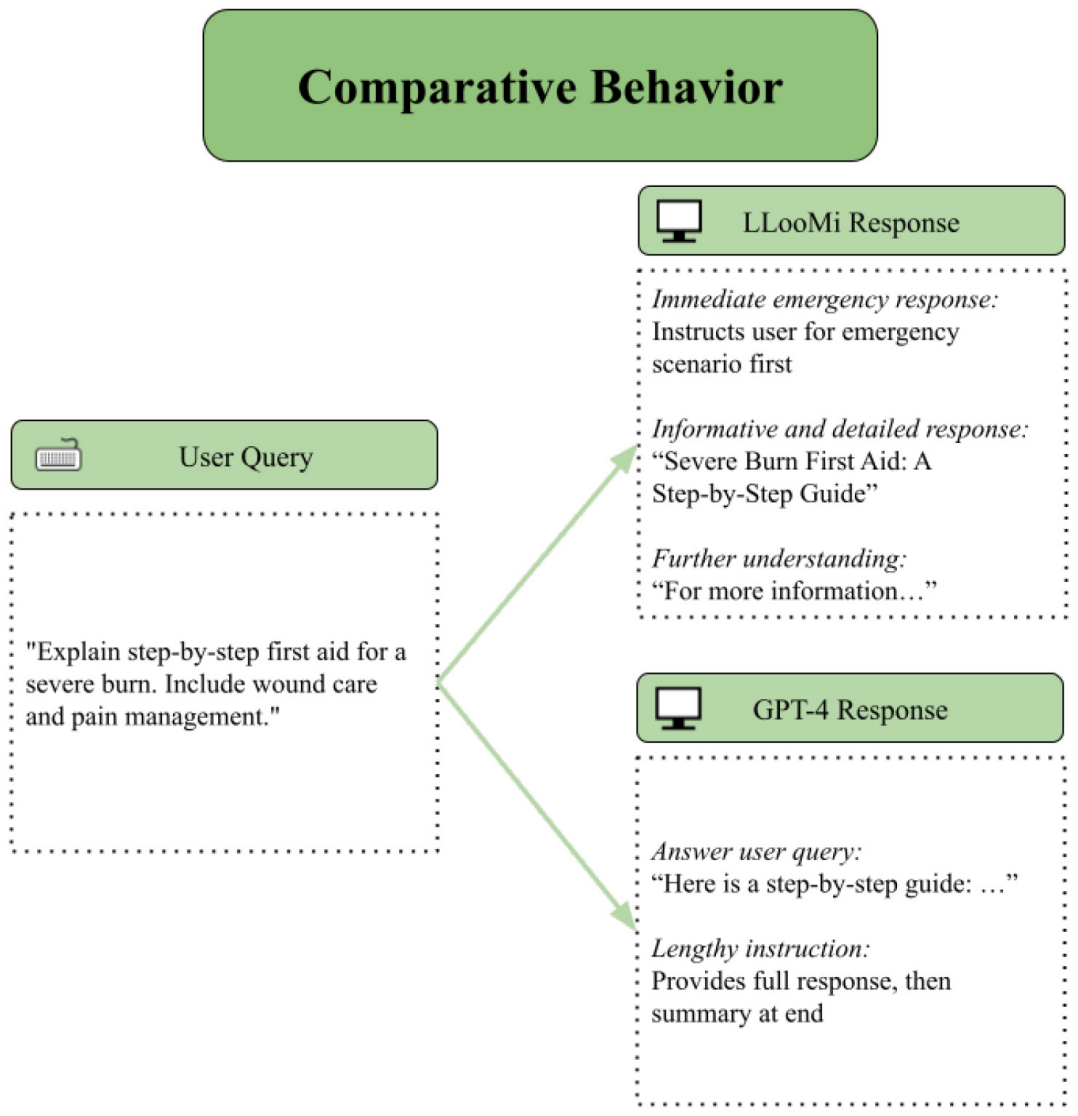
LLooMi's response in comparison to GPT-4, an off-the-shelf LLM.

In this case study, we examine LLooMi's behavior compared to OpenAI's GPT-4 model using a prompt from the medical domain. Figure 6 presents a user query asking for guidance on treating a minor burn injury, alongside LLooMi's generated response. This comparison evaluates factual coverage, clarity, empathy, and actionability, critical in high-stakes, user-facing digital health applications.

Both LLooMi and GPT-4 successfully enumerate the standard first-aid steps for burn care: cooling the burn, avoiding ointments, protecting the area with a sterile covering, and monitoring for signs of infection.

However, LLooMi's response demonstrates a marked structure and explanatory depth advantage. It lists procedural steps and justifies each action, such as why cold water is recommended over ice or when escalation to professional medical care is necessary. This listing aligns with LLooMi's instruction-primed approach, which encourages the model to explain the reasoning behind recommendations and guide users through critical decision points.

Moreover, LLooMi's output integrates supplementary resources and official references at the end of the response, such as links to the American Red Cross or Department of Health guidelines, providing users with opportunities to explore more detailed information beyond the immediate interaction. In contrast, GPT-4's response is more generic and does not direct users toward verified sources, potentially limiting its long-term utility in real-world support contexts.

Importantly, LLooMi's tone is explicitly calibrated to be reassuring and supportive, particularly when acknowledging the user's concern and suggesting next steps. This tone directly results from LLooMi's domain-specific instruction priming, which encourages emotionally attuned phrasing without requiring external sentiment classifiers or fine-tuning. This affective alignment enhances trust and user satisfaction in critical use cases such as minor injuries or emerging emergencies.

Through this comparative example, we observe how LLooMi maintains parity with GPT-4 in baseline factual accuracy, while offering substantial improvements in explanatory reasoning, resource linkage, and empathetic tone, key dimensions for digital assistants operating in sensitive healthcare contexts.

### 4.3 Case study 3: contextual response

**Figure 7:**
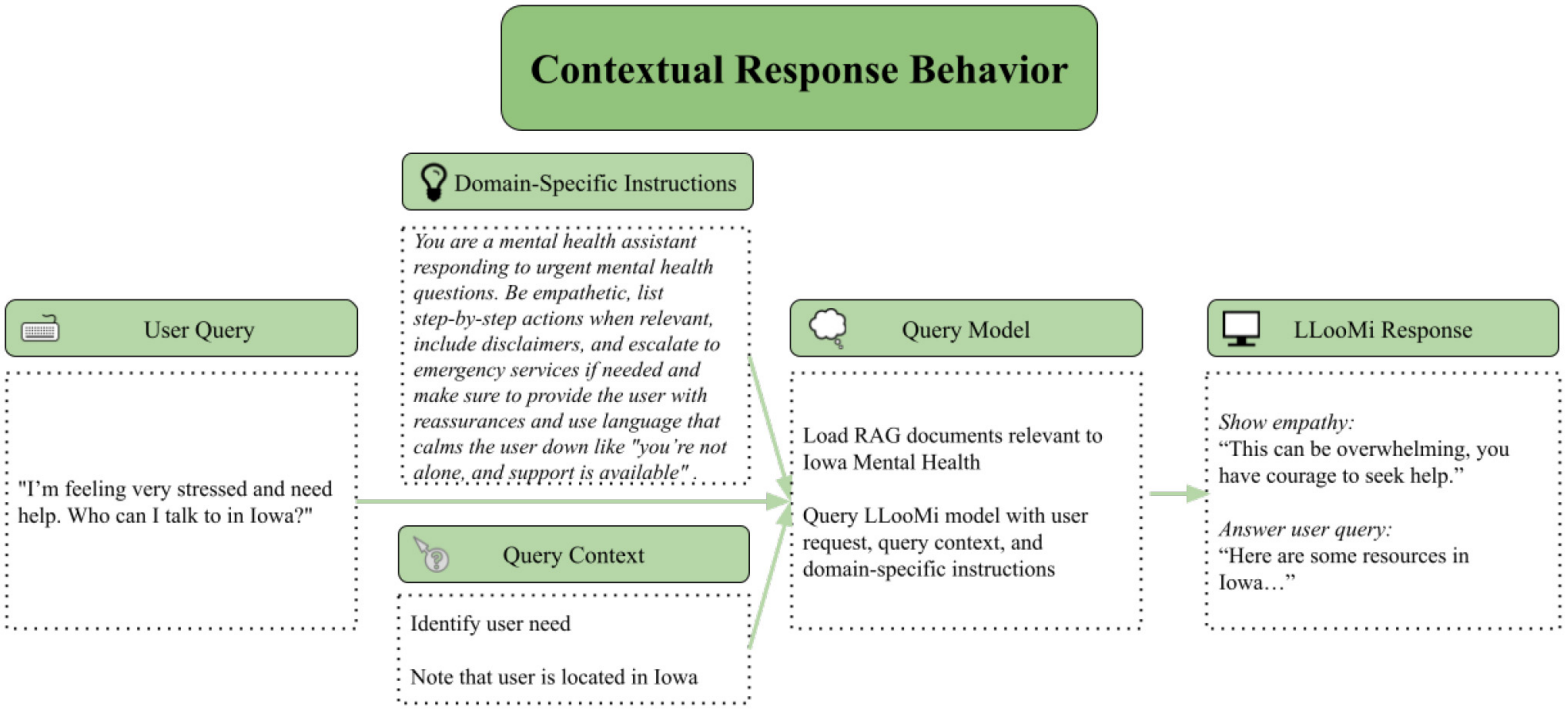
Illustration of LLooMi's contextual and empathetic response to a user's query.

**Figure 8:**
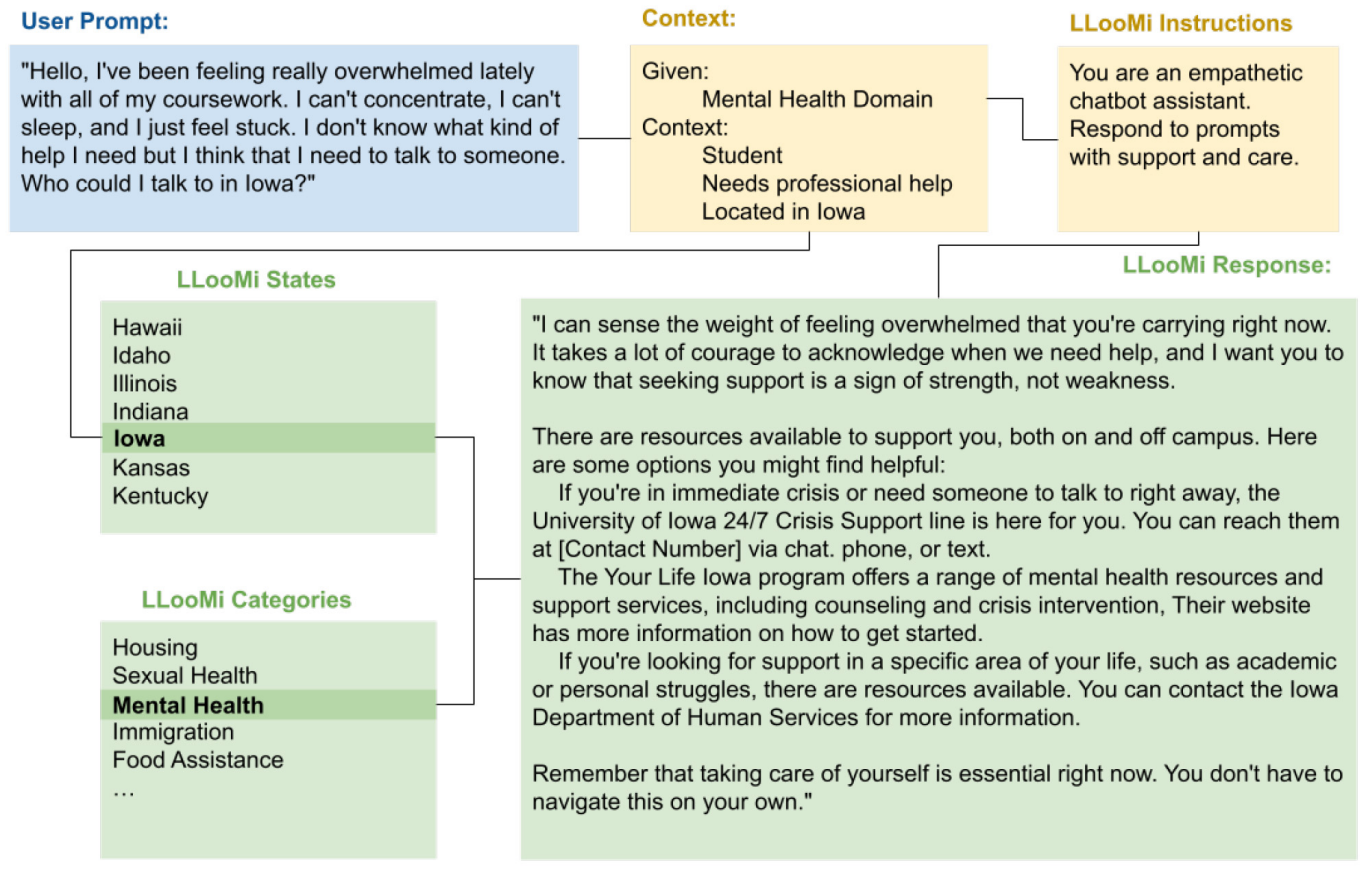
Details on how LLooMi uses chat context and given information to best pull relevant documents and respond to user queries.

For this case study, as seen in Figures 7, 8, and the domain topic used was mental health. The user is feeling overwhelmed, in need of support, and is looking for resources to point them in the direction of someone to talk to about their situation. Below is an example question from this distressed individual, along with the answer LLooMi provided them.

In Figure 7, LLooMi is given differing directions during training depending on the domain the user selects. Within mental health, LLooMi is directed to respond empathetically. In addition to this response, LLooMi should cater its response based on the context within the user's current or past queries within the same conversation. In this query and response, the user gives the additional request that the resources given are within Iowa. Figure 7 shows how LooMi is given directions specific to the domain of mental health and State of Iowa.

LLooMi responds to this question, as seen in Figure 8 beginning with an empathetic acknowledgment of the user's situation. This, in addition to the understanding tone and concise answer provided, presents the user with an empathetic response and sense of support. Furthermore, LLooMi successfully limits its recommendations to those within Iowa at the user's request.

### 4.4 Case study 4: unusual case

**Figure 9:**
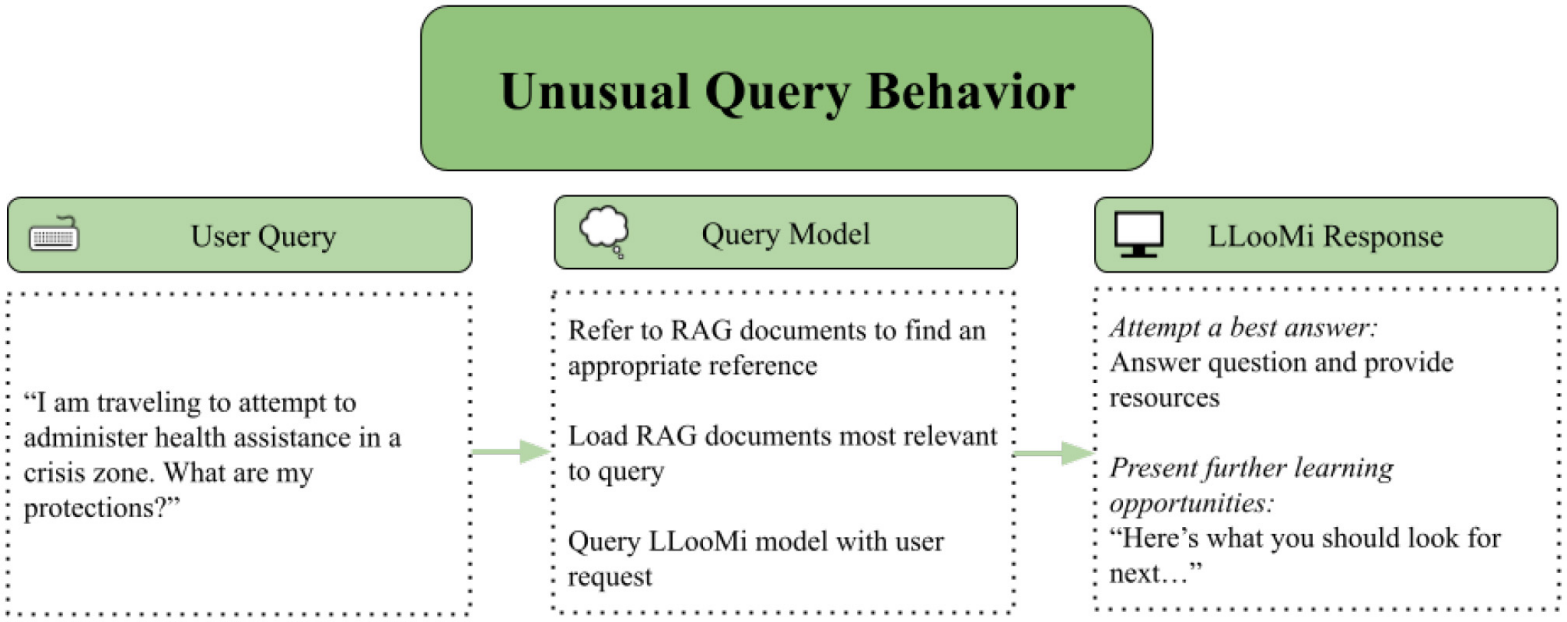
LLooMi processing and responding to a query outside its scope of context.

**Figure 10:**
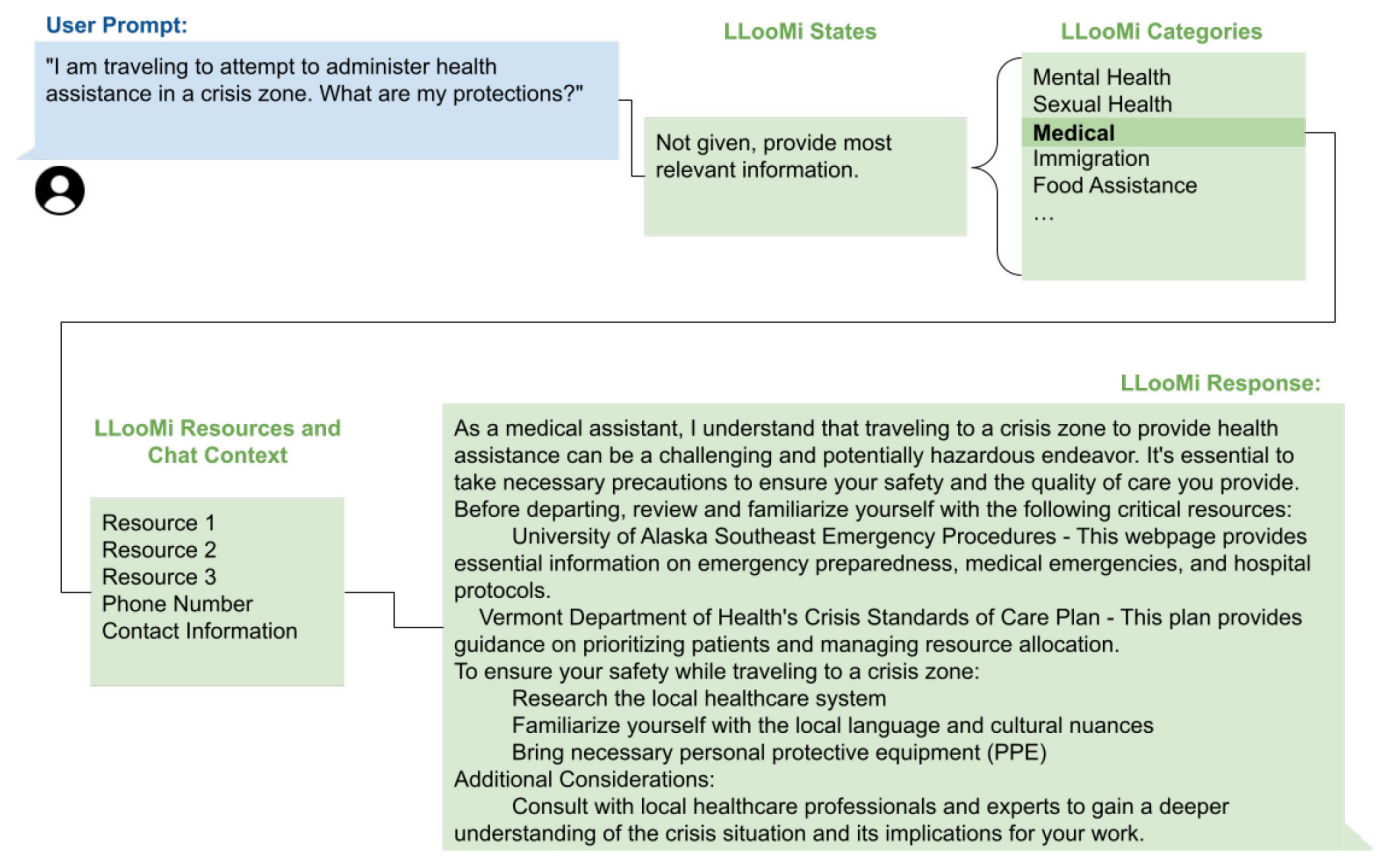
How LLooMi uses given documentation and its basic capabilities to give a relevant and complete answer to a query outside of its scope.

This case study demonstrates LLooMi's behavior when encountering a query outside its predefined geographic and domain scope. As illustrated in the internal flow of LLooMi and the response interactions—Figures 9, 10—the user requests guidance on administering healthcare in crisis zones, explicitly referring to international humanitarian contexts. However, LLooMi is currently trained on and grounded in curated knowledge bases focused exclusively on resources and infrastructure within the United States.

Upon receiving the query, LLooMi processes the conversation context and routes the request through its standard RAG pipeline. The history-aware retriever reformulates the query and performs semantic search within the U.S.-based medical and emergency response domains. Because no direct match for international crisis zone health protocols exists in the knowledge base, LLooMi invokes its fallback strategy: generating a generalized response based on best practices available within its accessible corpus.

Rather than producing an irrelevant or hallucinated answer, LLooMi acknowledges the query's implicit scope while shifting the response toward actionable preparedness guidelines, such as disaster triage principles, emergency kit assembly, and recommended training for crisis responders. It also suggests what types of region-specific information the user might seek, such as WHO field manuals, MSF guidelines, or local Ministry of Health advisories.

This case underscores LLooMi's capacity for graceful degradation when confronted with out-of-distribution inputs. Although the assistant cannot fulfill the query with full specificity, it still produces a contextually relevant and safety-conscious response, ensuring that the user is not misled and is instead redirected toward appropriate next steps. Importantly, the system does not attempt to hallucinate country-specific health information and maintains transparency in the scope of its assistance base.

Ideally, LLooMi would have access to globally scoped, multilingual datasets and localized resources. However, this case illustrates that the system upholds its core design values even under constrained retrieval conditions: providing verifiable information, maintaining an empathetic tone, and transparently communicating limitations. The interaction demonstrates LLooMi's robustness in ambiguous or edge-case scenarios and highlights the importance of incorporating broader knowledge domains and regional expansions in future work.

## 5 Discussion

LLooMi illustrates the promise of intent-aware LLMs as digital companions in high-stakes humanitarian and health-related scenarios. By grounding responses in structured, domain-specific knowledge and adapting to user intent across a wide range of distress categories, LLooMi demonstrates how conversational AI can offer practical assistance and emotional support. Our experience developing LLooMi revealed several key challenges and opportunities: the importance of maintaining up-to-date, regionally diverse data sources, the need for faster, more adaptive retrieval methods, and the benefits of integrating geolocation and multi-modal input streams to improve situational precision. Moreover, expanding coverage to additional help categories and improving latency for real-time use remain important areas for future refinement. These reflections reinforce LLooMi's broader objective as a user-centered, trustworthy companion for navigating moments of uncertainty or crisis.

### 5.1 Challenges

Throughout the development of LLooMi, several technical, operational, and ethical challenges emerged, necessitating iterative refinements across the retrieval, generation, and deployment pipelines.

A persistent and high-priority concern involved the generation of hallucinated outputs, instances in which the language model produced fabricated, factually incorrect, or contextually irrelevant content. Early in development, when evaluating out-of-the-box LLMs, the system exhibited what Huang et al. classify as faithfulness hallucinations (Huang et al., 2025), wherein generated content misrepresented or invented details from referenced material. Upon incorporating curated datasets and structured prompting into LLooMi's architecture, the model exhibited factuality hallucinations, where plausible but invented facts were introduced during attempts to fulfill a user query. These were mitigated through the adoption of a RAG pipeline, tuning of inference hyperparameters (e.g., temperature, frequency/presence penalties, max token limits), and refinement of domain-specific prompt templates to increase grounding and reduce stochastic variability in model behavior.

Another recurring issue involved abrupt or truncated outputs, where generated responses stopped mid-sentence or omitted critical steps. This issue resulted from token budget overflows or premature termination in autoregressive decoding. Token usage was systematically audited across the system prompt, conversation history, and retrieved documents, and output length constraints were increased where feasible. In addition, a lightweight post-processing step was introduced to detect sentence fragments and optionally re-trigger generation to complete the response.

From a systems standpoint, resource constraints presented a significant bottleneck, particularly during inference on virtual machines with limited VRAM and CPU memory. Out-of-memory (OOM) errors occurred frequently during multi-threaded document retrieval or concurrent user sessions. This challenge was mitigated by migrating to optimized LLM backbones (e.g., LLaMa 3.2B), dynamically adjusting batch sizes and retrieval depth, and implementing a least-recently-used (LRU) caching strategy for memory-efficient document index management. This design enabled domain-specific vector stores to be unloaded when inactive, reducing peak memory usage without sacrificing interactivity or response quality.

A related challenge was the limited granularity and completeness of publicly available datasets for humanitarian support tasks. Existing datasets often lacked regional specificity, up-to-date contact information, or multilingual considerations. To compensate, extensive manual curation was performed to extract and structure data from government portals, nonprofit directories, and trusted regional sources across eight domains. While labor-intensive, this approach improved document retrieval accuracy and reduced model dependence on generalized web knowledge.

From an ethical and compliance perspective, handling sensitive queries, particularly those concerning mental health, legal status, sexual health, or housing insecurity, requires careful alignment with principles of responsible AI. LLooMi was designed to operate without storing or logging personally identifiable information (PII) and without persisting conversational history across sessions. To support compliance with privacy standards such as HIPAA, GDPR, and NIST AI RMF, we embedded disclaimers throughout the interface, provided clear usage boundaries, and prevented the generation of prescriptive medical or legal advice.

Despite LLooMi's intent-aware architecture and grounding via semantic retrieval, ensuring response accuracy in high-stakes domains, especially mental health, remains a persistent challenge. LLMs, even when equipped with curated context, can produce hallucinated, outdated, or misaligned outputs that may inadvertently mislead users. To mitigate this, LLooMi implements system-level disclaimers presented before and during usage, explicitly informing users that the assistant may generate incorrect or incomplete information. Each interaction is prefaced with a safety reminder that LLooMi is not a substitute for professional care, and users facing urgent or life-threatening crises are strongly advised to contact local emergency services (e.g., 911 in the U.S.) or mental health crisis hotlines such as the Suicide and Crisis Lifeline (988). While the system is optimized for relevance, factuality, and empathy, it is critical to acknowledge the limitations of automated responses, especially in emotionally vulnerable contexts. This transparent warning mechanism reflects our commitment to responsible AI deployment in digital psychiatry and humanitarian assistance.

Another substantive challenge arose from intent ambiguity and misclassification. Despite LLooMi's instruction-primed framework for intent-aware response generation, there were instances where the LLM misinterpreted user urgency or misunderstood emotionally loaded queries. For example, emotionally neutral wording may have masked critical distress signals, resulting in overly generic or delayed responses. Conversely, emotionally expressive but informational queries were sometimes misclassified as crises, triggering overly terse or crisis-optimized outputs. Addressing this limitation remains ongoing, including incorporating lightweight intent classifiers and refining few-shot exemplars in prompt construction to better distinguish between high-urgency, low-context, and reassurance-seeking interactions.

Lastly, real-time response latency continues to impact user experience, especially in low-resource or bandwidth-constrained environments. Although retrieval and generation components are independently optimized, end-to-end system delay remains a bottleneck. To address this, future directions include local inference via quantized models, asynchronous retrieval pipelines, and caching of high-frequency question templates to pre-compile common responses and improve responsiveness.

### 5.2 Conclusions

In this work, we presented LLooMi, a retrieval-augmented, empathy-aware digital assistant designed to support users across various health and humanitarian domains. LLooMi demonstrates how LLMs can be deployed responsibly in high-stakes, emotionally sensitive contexts by integrating structured knowledge bases, instruction-primed response generation, and modular system design. Our evaluation highlights LLooMi's ability to deliver accurate, relevant, and emotionally attuned responses while preserving transparency, user safety, and ethical alignment. Case studies further illustrate the assistant's robustness across typical, comparative, and out-of-scope scenarios. LLooMi is a foundational step toward scalable, inclusive, and context-aware digital health interventions, with future work focused on expanding multilingual coverage, integrating real-time sentiment inference, enabling multi-modal input, and supporting human-in-the-loop escalation for high-risk conversations.

### 5.3 Future work

While LLooMi currently offers reliable, context-aware support through a retrieval-augmented and instruction-primed LLM pipeline, several areas remain for expansion to improve emotional responsiveness, accessibility, personalization, and global relevance. Future iterations will explore the integration of advanced modules that enhance the assistant's capability to detect user sentiment, support multi-modal and multilingual inputs, personalize responses based on long-term interaction patterns, and introduce human-in-the-loop escalation for high-risk scenarios. These developments further align LLooMi with inclusive, ethically grounded, and user-centered digital health support principles.

#### 5.3.1 Sentiment-aware response modulation

While LLooMi currently relies on domain-specific prompt priming to simulate empathetic behavior, future iterations of the system will explore the integration of robust and inference sentiment analysis to enhance emotional awareness. Specifically, we aim to implement a lightweight, real-time sentiment inference module capable of directly detecting user sentiment (e.g., distress, frustration, and urgency) from conversational input. This module would operate independently of the LLM and augment the retrieval and generation pipeline by dynamically adjusting instruction prompts, retrieval priorities, and response tone. For example, a detected high-distress state could trigger a more cautious response template, prioritize crisis-related resources, or escalate the conversation to pre-configured emergency guidance flows.

Transformer-based sentiment classifiers [e.g., DistilBERT, RoBERTa fine-tuned on emotion corpora (Sanh et al., 2020; Liu et al., 2019)] and low-latency alternatives suitable for edge deployment will be explored to expand LLooMi's functionalities. Importantly, all sentiment analysis will remain inference-only and non-persistent, preserving user privacy while enabling more contextually attuned real-time interactions.

#### 5.3.2 Mutli-modal input schemes

To enhance user accessibility and expand the range of supported interactions, future iterations of LLooMi will incorporate multi-modal input capabilities. While the current system is text-only, future versions will support speech-to-text interfaces, allowing users to engage with the assistant using naturally spoken language. This feature is especially valuable for users with limited literacy or visual impairments or in hands-free environments such as shelters, mobile clinics, or crisis scenarios where typing may not be feasible.

We also envision integrating image-based inputs, such as uploading medical forms, ID documentation for immigration support, or photos of medications or symptoms. This versatility will enable LLooMi to identify medication instructions, verify document requirements, or connect users to the appropriate service pathways based on visual information.

From a technical standpoint, these enhancements will involve incorporating pre-processing modules [e.g., speech recognition models, optical character recognition (OCR), or vision-language encoders] and aligning their outputs with the RAG pipeline for downstream retrieval and response generation. Care will be taken to ensure privacy preservation, data minimization, and secure processing of sensitive user-uploaded content.

Multi-modal capabilities will make LLooMi more inclusive, accessible, and responsive, especially for users in marginalized, low-resource, or high-stress environments. By supporting more naturalistic forms of input, we aim to reduce barriers to access and further align LLooMi with the principles of equity-centered digital health.

#### 5.3.3 Adaptive personalization through long-term memory

LLooMi currently provides consistent and factually grounded assistance using static datasets and generalized prompts. However, real-world users often have recurring needs, such as regional preferences or accessibility constraints, that static models typically cannot accommodate. Future iterations of LLooMi can leverage a nuanced long-term memory module that enables adaptive personalization without compromising user privacy. This approach provides each instance of LLooMi to locally fine-tune aspects of the underlying LLM and append contextual facts through a GPT-based memory module through repeated interactions like preferred tone or regional context while maintaining LLooMi's commitment to non-invasive data handling. To preserve safety and robustness, all local updates will feature aggregate using differential privacy techniques such as tokenized encryption and are applied only to constrained, non-generative layers while omitting personally identifiable information. This memory aspect can then improve the retrieval component while adhering to the user's communication needs while upholding strong ethical guarantees.

#### 5.3.4 Multilingual and low-resource language support

In its current state, LLooMi's audience receives resources within the United States of America. Expanding the diversity of resources to a larger corpus of regions embodies a critical direction in exploring LLooMi's effectiveness across different user bases. Since LLoomi is an assistant whose primary goal is to be a resource hub for those requesting information when they need it, expanding to a more global and diverse user base by targeting many of the vulnerable populations, refugees, displaced, and remote rural communities with low language resources as well as Indigenous languages that are poorly supported by present-day LLMs is a critical direction. LLooMi can be extended with the provision for multilingual assistance by incorporating language-agnostic embedding models and employing cross-lingual retrieval techniques.

For low-resource languages, datasets and inforamtion from humanitarian NGOs and local translators can be extracted to build special bilingual corpora that could later be used for fine-tuning and retrieval grounding while creating task-specific agents that can also automate and facilitate these steps. These agents and external outsource mechanisms can then be validated with language detection mechanisms and fallback translations to ensure that situations, where direct language support is unavailable, are addressed appropriately. For example, when LLooMi has access to agents that bypass the language barrier, interactions between users and resource bases of different languages and standards can easily be conveyed with language mechanisms and agents that address these issues. LLoMi would then grow this multilingual expansion as a cornerstone for the fulfillment and growth of LLooMi's intention of inclusive and accessible digital assistance.

#### 5.3.5 Contextual escalation with human-in-the-loop systems

Although LLooMi is designed to provide an emotionally aware and factually correct support system, we recognize the limitations of automated systems in properly dealing with high-risk or ethically sensitive conversations. Hence, in the future, LLooMi could implement a mechanism for contextual handoff, allowing it to route certain users to a qualified professional. Such queries would include those involving suicidal intent, abuse, or legal crisis, which would be flagged using intent classification and confidence scoring models. When a risk threshold is reached, LLooMi can negotiate a smooth handoff to certified crisis counselors, social workers, or live chat systems, with the conversation context preserved and the user's transparency maintained throughout. This hybrid approach would greatly enhance LLooMi's ability to offer support in acute distress situations while preserving accountability and safety in all other uses.

## Data Availability

The original contributions presented in the study are publicly available. This data can be found here: https://docs.google.com/spreadsheets/d/1RVEHrNbjzsA1GSPxbvHDAE-yhzUSvu0LZlaD0jUw-RE/edit?usp=drivesdk.

## References

[B1] Bhat S. R. Rudat M. Spiekermann J. Flores-Herr N. (2025). Rethinking chunk size for long-document retrieval: a multi-dataset analysis. arXiv preprint arXiv:2505.21700.

[B2] Brown T. Mann B. Ryder N. Subbiah M. Kaplan J. D. Dhariwal P. . (2020). “Language models are few-shot learners,” in Advances in Neural Information Processing Systems.

[B3] Celik A. Eltawil A. M. (2024). At the dawn of generative AI era: a tutorial-cum-survey on new frontiers in 6G wireless intelligence. IEEE Open J. Commun. Soc. 5, 2433–2489. doi: 10.1109/OJCOMS.2024.3362271

[B4] Chang T. A. Bergen B. K. (2024). Language model behavior: a comprehensive survey. Comput. Ling. 50, 293–350. doi: 10.1162/coli_a_00492

[B5] Chen H. Liu X. Yin D. Tang J. (2017). A survey on dialogue systems: recent advances and new frontiers. CoRR, abs/1711.01731.

[B6] Chen N. Liu J. Dong X. Liu Q. Sakai T. Wu X.-M. (2024). “AI can be cognitively biased: an exploratory study on threshold priming in LLM-based batch relevance assessment,” in Proceedings of the 2024 Annual International ACM SIGIR Conference on Research and Development in Information Retrieval in the Asia Pacific Region, SIGIR-AP 2024 (New York, NY, USA: Association for Computing Machinery), 54–63. doi: 10.1145/3673791.3698420

[B7] Douze M. Guzhva A. Deng C. Johnson J. Szilvasy G. Mazar P.-E. . (2024). The FAISS library. IEEE Trans. Big Data. 2025, 1–17. doi: 10.1109/TBDATA.2025.3618474

[B8] Dubey A. Jauhri A. Pandey A. Kadian A. Al-Dahle A. Letman A. . (2024). The llama 3 herd of models. arXiv e-prints, arXiv-2407.

[B9] Gao Y. Xiong Y. Gao X. Jia K. Pan J. Bi Y. . (2023). Retrieval-augmented generation for large language models: a survey. arXiv preprint arXiv:2312.10997.

[B10] Großmann G. Ivanova L. Poduru S. L. Tabrizian M. Mesabah I. Selby D. A. . (2025). The power of stories: narrative priming shapes how LLM agents collaborate and compete. arXiv preprint arXiv:2505.03961.

[B11] Huang L. Yu W. Ma W. Zhong W. Feng Z. Wang H. . (2025). A survey on hallucination in large language models: principles, taxonomy, challenges, and open questions. ACM Trans. Inf. Syst. 43, 1–55. doi: 10.1145/3703155

[B12] Kraljevic Z. Shek A. Bean D. Bendayan R. Teo J. T. Dobson R. J. B. (2021). Medgpt: Medical concept prediction from clinical narratives. CoRR, abs/2107.03134.

[B13] Lewis P. Perez E. Piktus A. Petroni F. Karpukhin V. Goyal N. . (2020). “Retrieval-augmented generation for knowledge-intensive NLP tasks,” in Proceedings of the 34th International Conference on Neural Information Processing Systems, NIPS '20 (Red Hook, NY, USA: Curran Associates Inc.).

[B14] Liu Y. Ott M. Goyal N. Du J. Joshi M. Chen D. . (2019). RoBERTa: a robustly optimized BERT pretraining approach. arXiv preprint arXiv:1907.11692.

[B15] Maharana K. Mondal S. Nemade B. (2022). A review: data pre-processing and data augmentation techniques. Global Transit. Proc. 3, 91–99. doi: 10.1016/j.gltp.2022.04.020

[B16] Mayer H. Richards J. (2025). Comparative analysis of distributed caching algorithms: performance metrics and implementation considerations. arXiv preprint arXiv:2504.02220.

[B17] Plaat A. van Duijn M. van Stein N. Preuss M. van der Putten P. Batenburg K. J. (2025). Agentic large language models, a survey. arXiv preprint arXiv:2503.23037.

[B18] Prasetyo P. K. Achananuparp P. Lim E.-P. (2020). “Foodbot: a goal-oriented just-in-time healthy eating interventions chatBot,” in Proceedings of the 14th EAI International Conference on Pervasive Computing Technologies for Healthcare, PervasiveHealth–20 (ACM). doi: 10.1145/3421937.3421960

[B19] Radford A. Wu J. Child R. Luan D. Amodei D. Sutskever I. (2019). Language models are unsupervised multitask learners. OpenAI Blog 1:9. Available online at: https://api.semanticscholar.org/CorpusID:160025533 (Accessed July 12, 2025).

[B20] Rajkomar A. Dean J. Kohane I. (2019). Machine learning in medicine. New Engl. J. Med. 380, 1347–1358. doi: 10.1056/NEJMra1814259 30943338

[B21] Sanh V. Debut L. Chaumond J. Wolf T. (2020). DistilBERT, a distilled version of BERT: smaller, faster, cheaper and lighter. arXiv preprint arXiv:1910.01108.

[B22] Thirunavukarasu A. J. Ting D. S. J. Elangovan K. Gutierrez L. Tan T. F. Ting D. S. W. (2023). Large language models in medicine. Nat. Med. 29, 1930–1940. doi: 10.1038/s41591-023-02448-8 37460753

[B23] World Health Organization (2022). Healthbuddy+: *Access to Trusted Information on COVID-19 in Local Languages Using an Interactive Web- and Mobile-Based Application*. Geneva: World Health Organization.

[B24] Xu Z. Jain S. Kankanhalli M. (2024). Hallucination is inevitable: an innate limitation of large language models. ArXiv, abs/2401.11817.

[B25] Zhang D. Li J. Zeng Z. Wang F. (2025). Jasper and Stella: distillation of SOTA embedding models. arXiv preprint arXiv:2412.19048.

[B26] Zheng Z. Ning K. Wang Y. Zhang J. Zheng D. Ye M. . (2023). A survey of large language models for code: evolution, benchmarking, and future trends. arXiv preprint arXiv:2311.10372.

